# Association Between Obesity and Lower Urinary Tract Symptoms Among Children and Adolescents: A Community-Based Study

**DOI:** 10.3389/fped.2021.609057

**Published:** 2021-04-13

**Authors:** Shih-Gang Wang, Stephen Shei-Dei Yang, Shang-Jen Chang

**Affiliations:** ^1^Division of Urology, Department of Surgery, Taipei Tzu Chi Hospital, The Buddhist Tzu Chi Medical Foundation, Taipei, Taiwan; ^2^School of Medicine, Tzu Chi University, Hualien, Taiwan

**Keywords:** lower urinary tract symptoms, urgency, incontinence, dysfunctional voiding symptom score, children, adolescents, obesity

## Abstract

**Introduction:** Obesity is associated with lower urinary tract symptoms (LUTSs) and dysfunction in adults while its impact on children and adolescents remains unknown. This study aimed to explore the impact of obesity on LUTSs among children and adolescents through a large-scale community-based study.

**Methods:** From July 2004 to April 2017, children and adolescents aged 5–15 years-old in Xin-Dian District, New Taipei City were invited to participate in our study. The exclusion criteria were a history of congenital genitourinary tract anomalies, neurological anomalies, or a presence of urinary tract infection. After providing informed consent the participant completed a questionnaire, which included their baseline characteristics and dysfunctional voiding symptom score (DVSS); a parent completed the questionnaire with the younger children. Urgency and daytime incontinence were defined as having positive statement for DVSS questions 7 and 1, respectively. Multivariate regression analysis was used to evaluate the predictors of urgency, daytime incontinence and enuresis. A *p*-value of <0.05 was considered statistically significant.

**Results:** A total of 2,371 participants were enrolled in the study, and 1,599 were ultimately eligible for analysis. The prevalence of urgency, daytime incontinence, constipation, and enuresis were 37.6, 6.4, 26.1, and 7.7%, respectively. Multivariate analysis revealed that younger age (*p* = 0.01) and obesity (*p* = 0.04) were independent predictors for urgency. Younger age (*p* < 0.01) and constipation (*p* = 0.04) were independent predictors for daytime incontinence but obesity was not. Younger children were more likely to have nocturnal enuresis (95% CI = 0.77–0.88) and obesity did not have a significant impact on enuresis.

**Conclusion:** Obesity was significantly associated with urgency but it was not significantly associated with daytime incontinence and enuresis in community dwelling children and adolescents.

## Introduction

Obesity has become a major public health issue in the modern era and its prevalence has increased several fold over the past few decades (1, 2). Obese children are known to have an increased risk of fracture and are at higher risk of having adulthood obesity, premature death, hypertension, early markers of cardiovascular disease, insulin resistance, and psychological effects (3). Obesity is also associated with bladder dysfunction. The underlying pathophysiology of how obesity contribute to lower urinary tract dysfunction and symptoms has not yet been clarified. Obesity has been previously shown to be associated with lower urinary tract symptoms (LUTSs) in adulthood (4–6). According to a previous study by the authors that included 838 children (aged 8.0 ± 2.0 years), obesity is associated with higher urgency. However, data regarding the prevalence of LUTSs in adolescents and their association with obesity remains scarce. Therefore, the current study included more adolescents and children to further illustrate the prevalence of LUTS in children and adolescents aged 5 to 15 years old, and to investigate the potential association between obesity and LUTSs.

## Methods

The current study was an observational study which was approved by the Institutional Review Board at Taipei Tzu-Chi Hospital in New Taipei City of Taiwan. Informed consent was obtained from all participants when they were enrolled into the study. Between July 2004 and April 2017, students from the elementary schools and junior high schools in Xin-Dian District, New Taipei City were invited to participate in this study. Individuals were excluded from the study if they had congenital genitourinary tract anomalies, neurological anomalies, or a history of urinary tract infection. The participants took urinary dipsticks tests when they were enrolled into the study and if they had a positive leukocyte esterase or nitrites result on the test, they were also excluded from the analysis. In summary, the inclusion criteria of our study were (1) aging 5–15 years old, and (2) receiving at least one uroflowmetry test. The exclusion criteria of the study were (1) congenital genitourinary tract anomalies, (2) neurologic anomalies, (3) recurrent or active urinary tract infection, and (4) incomplete answer of the questionnaire.

### Definition

Body mass index (BMI) is defined as the individual's weight (kg) divided by the square of their height (m^2^). The participants were divided into normal weight, overweight, and obese group as determined by the definition given in the Children and Adolescents Growth References, Department of Health, Executive Yuan, Republic of China, published in June 2013. The participants were considered as overweight if their BMI was between the 85th and 95th percentile on gender- and age-specific nomograms. If the participants BMI was over the 95th percentile on gender- and age-specific nomograms, they were considered as obese.

### Questionnaire

Elementary school children aged 5–15 years old completed a questionnaire with one of their parents that included their baseline characteristics (age, gender, body weight, and height), presence of snoring or sleep apnea during the past month, dysfunctional voiding symptom score (DVSS), status of nocturnal enuresis (defined as “having at least one episode of wetting in discrete portions while asleep in a child who has passed his or her fifth birthday in the past 1 month”). Junior high school adolescents completed the questionnaire by themselves. The body height and weight of the participants were measured at health check-up of semester and reported by parents or the participants themselves.

Of the 2,371 participants invited to join our study, 1,599 (67.4%) children were finally included for analysis. The DVSS has 10 sections, including seven questions about LUTSs (daytime incontinence, amount of incontinence, infrequent voiding, curtsying, urgency, push to void, and dysuria), two questions about bowel movement (low defecation frequency and difficult defecation), and one question about stressful events (new home, new baby, new school problem, abuse, accidents, home problem, or another special event in the past month). The Chinese version of the DVSS was validated in children with dysfunctional voiding (7). Urgency and daytime incontinence symptom were assessed according to the statement, “When I have to pee, I cannot wait” and “I have had wet clothes or wet underwear during the day,” respectively. The symptoms were scored on a scale of 0–3 (0, almost never; 1, less than half of the time; 2, about half of the time; and 3, almost every time). We defined urgency and daytime incontinence as a symptom score of 1, or more than 1, respectively. Defecation conditions and stool form were assessed using the Bristol Stool Form Scale (8). If the answer was type 1 or type 2, the children and adolescents were categorized as having constipation.

### Statistical Analysis

Data were expressed as the mean ± standard deviation and analyzed using commercial statistical software (Medcalc1, version 12.7, Software bvba, Ostend, Belgium, and SAS1, version 9.2, SAS Institute, Inc., Cary, NC, USA). Demographic and clinical parameters were compared using an analysis of variance model (continuous demographic variables) and a Chi-squared test (categorical data) and a Kruskal–Wallis test (ordinal data). A Cochran–Armitage trend test was used to evaluate the trend in daytime incontinence and overactive bladder (OAB: defined as a sudden or uncontrollable urge to urinate) proportion across age. A multivariate regression analysis model was used to evaluate the predictors (age in years, gender, non-obese vs. obese, constipated vs. non-constipated) for urgency, daytime incontinence, and nocturnal enuresis. The risk factors were calculated as odds ratios (ORs) with 95% confidence intervals (95% CIs) for enuresis. A *P*-value of <0.05 was considered statistically significant.

## Results

**Figure 1:**
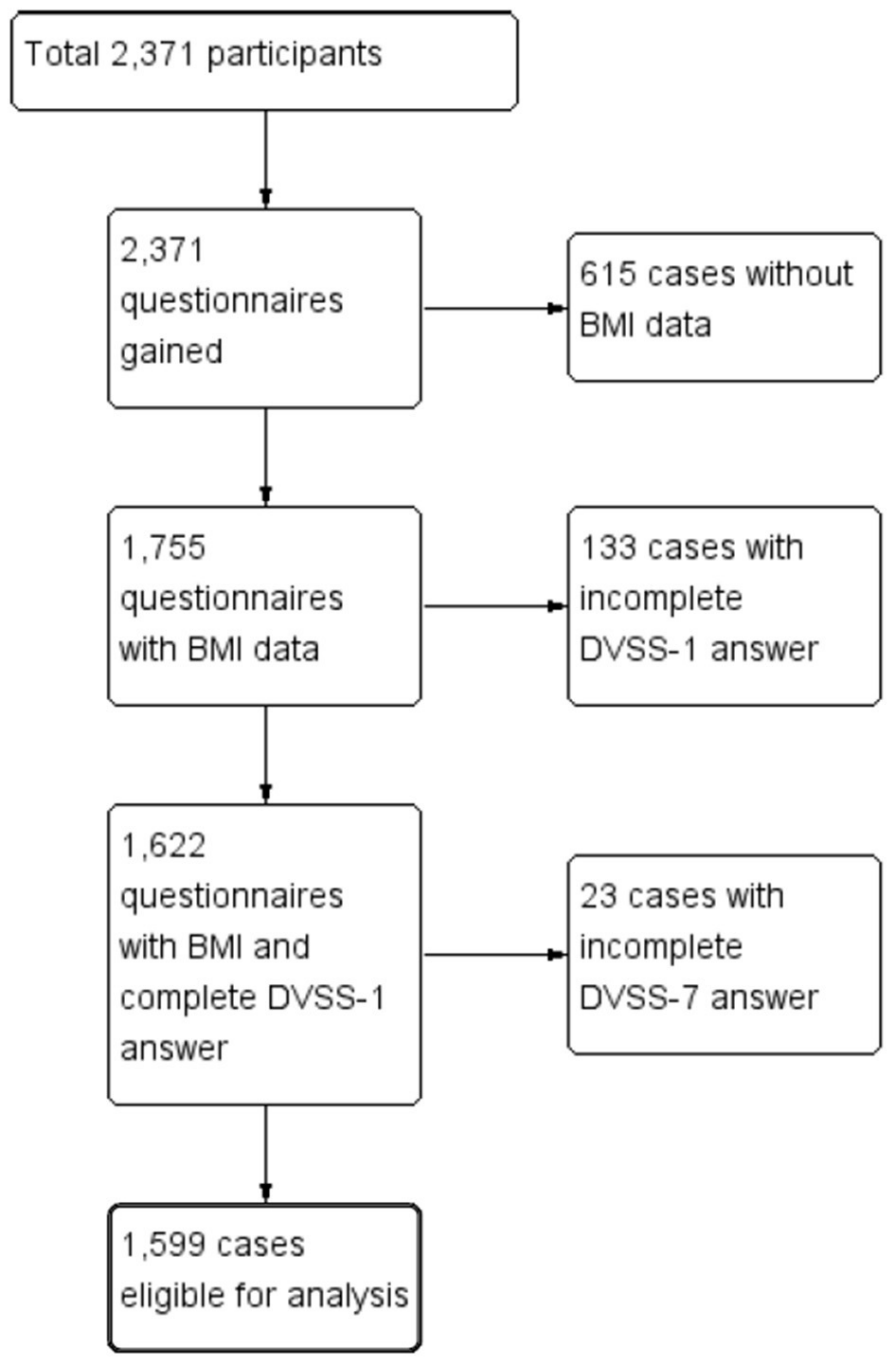
Flow chart of participant inclusion.

Figure 1 is a flow chart of the participants included within the study. A total of 2,371 questionnaires were distributed to the participating schools. After we excluded the children who did not meet the inclusion criteria and or had provided incomplete answers to the questionnaire, a total of 1,599 children (769 boys vs. 830 girls, age: 10.04 ± 3.66 years vs. 10.39 ± 3.95 years, *p* = 0.06) were eligible for analysis (the case numbers in each subgroup: 5–7 year-old = 465, 8–10 year-old = 360, 11–13 year-old = 360, and 14–15 year-old = 414). Table 1 gives a comparison of the baseline characteristics and LUTSs between the boys and the girls. The prevalence of obesity was significantly higher in the boys (15.3%) compared with the girls (9.6%, *p* < 0.01). The rate of constipation was significantly higher in the girls (30.3%) compared with the boys (21.6%, *p* < 0.01). Table 2 details the prevalence of incontinence, urgency, and constipation between the non-obese and obese groups. The obese group was older than the non-obese group and the prevalence of obesity was higher in the boys. Other risk factors, including urgency, constipation, and incontinence showed no significant differences between these two groups.

**Table 1:** Average age and prevalences for obesity, incontinence, urgency, and constipation in boys and girls.

	**Boys (*n* = 769)**	**Girls (*n* = 830)**	***p*-value**
Age (years, mean ± S.D)	10.04 ± 3.66	10.39 ± 3.95	0.06
Body height (cm, mean ± S.D)	140.88 ± 0.15	139.45 ± 0.14	0.50
Body weight (kg, mean ± S.D)	39.49 ± 0.46	37.42 ± 0.42	<0.01
Obese (95% CI)	15.3% (12.9~18.1%)	9.6% (7.7~11.9%)	<0.01
Incontinence (95% CI)	7.2% (5.4~9.2%)	5.7% (4.2~7.5%)	0.22
Urgency (95% CI)	37.50% (34.4~41.1%)	37.8% (34.5~41.2%)	0.90
Constipation (95% CI)	21.6% (18.8~24.7%)	30.3% (27.1~33.6%)	<0.01

**Table 2:** Prevalences for incontinence, urgency, and constipation in non-obese and obese group.

	**Non-obese (*n* = 1,401)**	**Obese (*n* = 198)**	***p*-value**
Age (Mean ± S.D)	10.11 ± 3.83	10.98 ± 3.60	<0.01
Boys (95% CI)	46.5% (43.8~49.1%)	59.6% (52.4~66.5%)	<0.01
Incontinence (95% CI)	6.6% (5.3~8.0%)	5.1% (2.4~9.1%)	0.41
Urgency (95% CI)	37.0% (34.5~39.7%)	42.1% (35.0~49.3%)	0.18
Constipation (95% CI)	26.5% (24.2~28.9%)	23.4% (17.6~29.9%)	0.34

### Urgency

**Figure 2:**
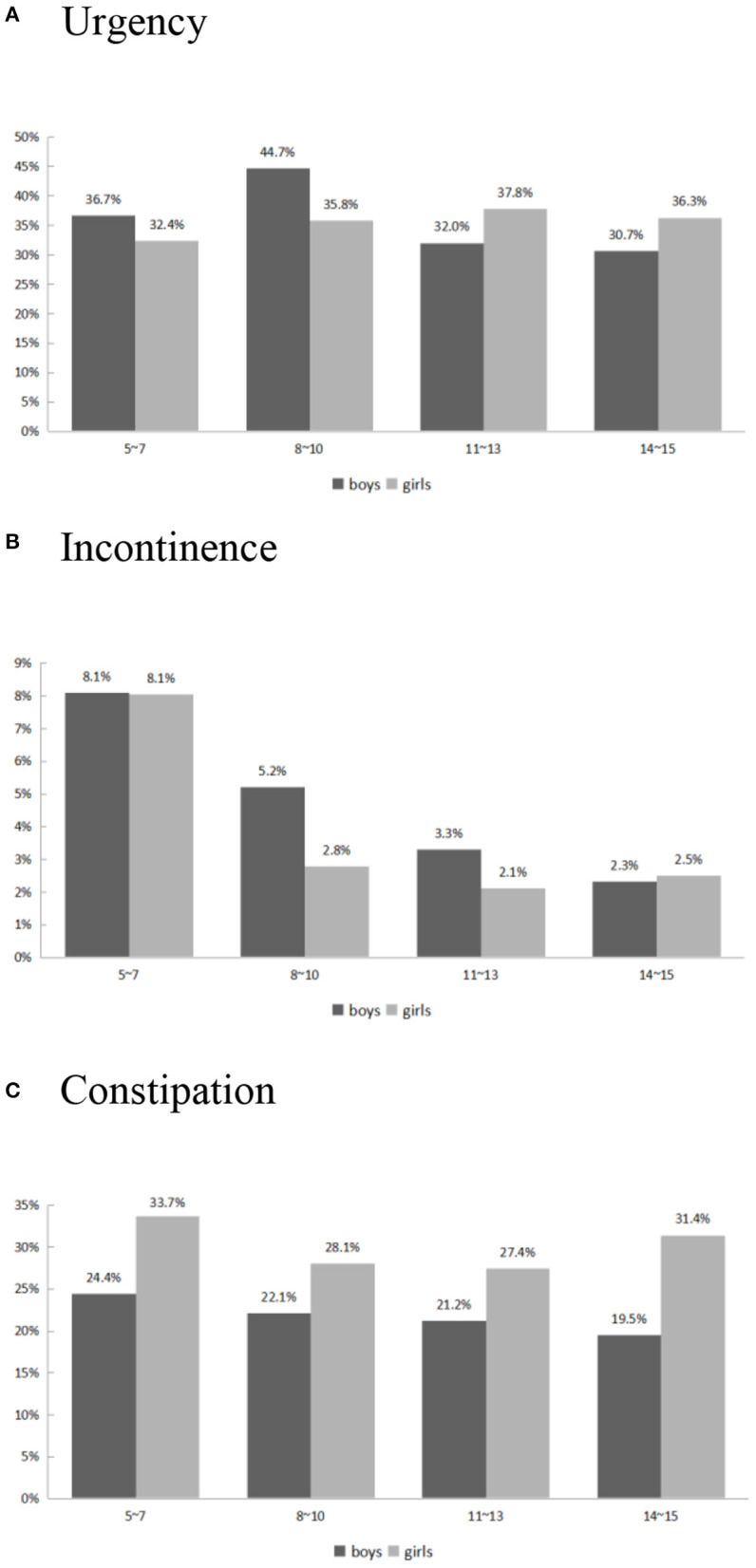
Self-reported Urgency **(A)**, Incontinence **(B)**, and Constipation **(C)** risk vs. age and gender.

The urgency score decreased from 0.55 ± 0.09 in 5–7 year-old children to 0.43 ± 0.08 in 14–15 year-old adolescents. Figure 2A depicts the prevalence of self-reported urgency (urgency score ≥1 in the DVSS-7) in our study population. There was no significant difference in self-reported urgency between genders (*p* = 0.63) or individuals with different constipation statuses (*p* = 0.53). The children and adolescents with obesity had a significantly higher risk of urgency compared with the non-obese group (*p* = 0.04). Younger age was also a significant predictor of a higher urgency scores (*p* = 0.01).

### Incontinence

The incontinence scores decreased from 0.10 ± 0.04 in 5–7 year-old children to 0.02 ± 0.02 in 14–15 year-old adolescents. The self-reported incontinence rate (incontinence score ≥1 in the DVSS-1) over variable ages is shown in Figure 2B. The significant risk factors for higher incontinence scores were younger age (*p* = 0.0002) and constipation (*p* = 0.04). Obesity and gender did not have a significant impact on incontinence in children and adolescents.

### Nocturnal Enuresis

The prevalence of nocturnal enuresis was 13.0% in 5–7 year-old children and 2.7% in 14–15 year-old adolescents. The only significant protective factor was older age (OR 0.82; 95% CI: 0.77–0.88). Gender, BMI, and constipation status were not significant risk factors.

### Constipation

The prevalence of constipation was 29.1% in 5–7 year-old children and 26.4% in 14–15 year-old adolescents (Figure 2C). The constipation rate was significantly lower in boys (OR: 0.63, 95% CI: 0.50–0.80) compared with girls. Age and obesity were not risk factors for constipation (OR 0.99; 95% CI: 0.96–1.02, and OR 0.88; 95% CI: 0.61–1.26, respectively).

## Discussion

The current study was a large-scale community-based study (*n* = 1,599) that explored the prevalence of LUTSs in community dwelling children and adolescents and evaluated their potential association with obesity. The prevalence of obesity was 12.7% among our study population. The prevalence of urgency, daytime incontinence, and enuresis in children aged 5–7 years old was 34.5, 8.0, and 13.0%, respectively. LUTSs decreased as age increased. The prevalence of urgency, daytime incontinence, and enuresis in adolescents aged 14–15 years was 33.8, 2.4, and 2.8%, respectively. The current study revealed that obesity was associated with higher urgency scores but not significantly associated with incontinence or nocturnal enuresis. The possible underlying pathophysiology of how obesity affects LUTSs and function are listed below.

The increasing number of LUTSs observed in obese children could be a consequence of hormonal, dietary, and even personality/behavioral differences. Children with obesity were observed to have more psychological distress than non-obese children (9–12). Obese children may suffered from increased intra-abdominal pressures which could result in the development of urinary urgency or incontinence (13). Additionally, obesity has also been considered to be associated with hyperglycemia (14). Hyperglycemia could cause diuresis, leading to LUTSs. There are several hypotheses that could explain the association between pediatric OAB and constipation. First, stool in the rectum stimulates stretch receptors which transmit a signal to the brain and induced the contraction of external anal sphincter and puborectalis muscles. Second, a distended rectum compresses the adjacent organs, such as the urinary bladder, which results in decreased capacity (15). Our current study confirmed that urgency was the core symptom of lower urinary tract dysfunction in obese children and adolescents. This finding also indicates the importance of body weight control in school-aged children. The benefits could not only be a reduction in cardiovascular disease, insulin resistance and musculoskeletal disorders but also less LUTSs.

Urgency is the core symptom of childhood OAB (16). The International Children Continence Society also mentioned other relative symptoms, including urge incontinence, urinary frequency, and nocturnal enuresis (17, 18). In the current study, we defined urgency and incontinence as when the urgency and incontinence symptom scores were ≥1. A former study by the authors demonstrated that childhood obesity was an independent risk factor for pediatric OAB symptoms (OR 1.97; 95% CI: 1.14–3.40) (14). Similarly, a large scale study in China which was conducted by Xing et al. (19) and evaluated 10,133 anonymous questionnaires, disclosed that children with obesity, a history of urinary tract infection, nocturnal enuresis, a family history of LUTSs, constipation, and fecal incontinence, had a significantly higher prevalence of OAB compare with normal children (*p* < 0.05). Fraga et al. (20) conducted a cross-sectional study which included 423 children and adolescents aged 5–17 years. There were three factors that were independently and significantly associated with a positive DVSS: age <10 years (95% CI: 0.34–1.18), constipation (95% CI: 0.88–2.70), and obesity (95% CI: 0.25–1.52). The author reported that only the bladder-filling symptoms of lower urinary tract dysfunction appeared to be associated with obesity. To further explore the impact of obesity on LUTSs among children and adolescents, we conducted current study. Multivariate regression analysis revealed that obesity was a significant independent risk factor for urgency (*p* = 0.04). Obese patients with severe OAB symptoms may warrant further examination for lower urinary tract dysfunction because both childhood OAB symptoms and obesity are considered to have a negative effect on the social, emotional, and behavioral development of children (12, 21). A previous cohort study also found that children with OAB symptoms were significantly more likely to have OAB symptoms in adulthood (9).

Obesity was also thought be associated with urinary incontinence in children. A Danish study conducted by Warner et al. (22) demonstrated an association between daytime urinary incontinence and BMI. Girls with one OAB symptom had a significantly higher average BMI than girls with no OAB (BMI-SDS 0.18 vs. 0.30 [*p* < 0.05] and 0.17 vs. 0.29 [*p* < 0.01], respectively). Obesity led to a significantly increased risk of nocturia in both adolescent boys and girls (OR 1.74; CI: 1.17–2.60 and OR 2.01; CI: 1.25–3.23, respectively). The study by Wagner et al. (23) also concluded that being overweight or obese gives you a significant higher risk of developing OAB symptoms, especially urinary incontinence (*p* = 0.017). A recent study by von Gontard et al. (24) supported the theory that behavioral symptoms, psychiatric disorders, and being overweight/obese were risk factors associated with urinary incontinence. However, we did not observe a significantly higher risk of daytime incontinence in obese children and adolescents in the current study. Our analysis indicated that younger age and constipation were the two independent risk factors for pediatric incontinence. A possible explanation for the non-significant impact of obesity on daytime incontinence could be the lower incontinence scores in the study populations, especially in the adolescents, (score: 0.03 ± 0.16 in 14–15 year-old adolescents). Also, the case number may not have been large enough to show the differences.

Erdem et al. (25) concluded that most of the children with voiding dysfunction were obese, and that their risk of developing the condition was almost double that of the normal population. A Chinese study by Ma et al. (26) also agreed that risk of severe monosymptomatic nocturnal enuresis (MNE) was higher in obese children. They also found that behavioral therapy was less effective in obese children with MNE. However, in our study, the prevalence of nocturnal enuresis was not significantly increased in the obesity group. We detected a higher nocturnal enuresis rate in younger children, which was compatible with current theories. According to a study from several decades ago, the prevalence of nocturnal enuresis decreases with age (27). Hamed et al. (28) performed a cross-sectional study, which included 4,652 school-age children in Egypt. The younger age categories showed a higher prevalence of MNE compared with the older age categories. The OR ranged from 6-year-olds (OR 3.17, 95% CI: 2.34–4.30) to 11-year-olds (OR 1.47, 95% CI: 1.04–2.09). Further studies needs to be conducted to clarify the association between children's BMI and nocturnal enuresis.

The current study had some limitations. First, in some cases the DVSS questionnaire was answered by the one parent along with the participating child. A bladder diary was not conducted and there may have been a discrepancy between a bladder diary and the questionnaire. Second, among the 2,371 participants, only 1,599 were eligible for inclusion within the study based on their questionnaire responses. A total of 772 children were excluded. The strict inclusion criteria may have resulted in the removal of some suitable cases from our study, which may have impacted the findings. Third, we defined the cut-off point of for urgency and daytime incontinence as having “1 or more” symptoms. However, the cut-off point for the definition of these conditions needs further investigation. The overall incontinence prevalence was 6.4%. The case number would be too scarce to analyze if we used “2 or more” cut-off point and low prevalence made it harder to reach significance in multivariate regression analysis model. The *p*-value was 0.94 in obese vs. non-obese and 0.33 in boy vs. girl group, separately. The study did have several strengths. It enrolled a total of 1,599 children and adolescents, which is a notable increase on the previous study which included only 838 children. We further separated LUTSs into urgency, incontinence and nocturnal enuresis. Each symptom had one corresponding question in DVSS. This is easier to analyze the data and to repeat similar studies in the future.

## Conclusion

The results of the current study revealed that obesity was significantly associated with urgency but not daytime incontinence and enuresis in community dwelling children and adolescents.

## Data Availability Statement

The raw data supporting the conclusions of this article will be made available by the authors, without undue reservation.

## Ethics Statement

The studies involving human participants were reviewed and approved by IRB No: 06-XD29-063. Written informed consent to participate in this study was provided by the participants' legal guardian/next of kin.

## Author Contributions

SY and S-JC: project development, data collection, and manuscript writing. S-GW and S-JC: manuscript writing. All authors contributed to the article and approved the submitted version.

## Conflict of Interest

The authors declare that the research was conducted in the absence of any commercial or financial relationships that could be construed as a potential conflict of interest.
